# Prevalence of Major Depressive Disorder Among Spouses of Men Who Use Alcohol in a Rural Community in Central Sri Lanka

**DOI:** 10.1093/alcalc/agu105

**Published:** 2015-01-14

**Authors:** Dewasmika Ariyasinghe, Ranil Abeysinghe, Prabhash Siriwardhana, Tharaka Dassanayake

**Affiliations:** 1 Department of Psychiatry, Faculty of Medicine, University of Peradeniya, Peradeniya, Sri Lanka; 2 South Asian Clinical Toxicology Research Collaboration, Faculty of Medicine, University of Peradeniya, Peradeniya, Sri Lanka; 3 Department of Physiology, Faculty of Medicine, University of Peradeniya, Peradeniya, Sri Lanka

## Abstract

**Aims:** To estimate the prevalence of major depressive disorder (MDD) among spouses of men who use alcohol in two rural areas in Sri Lanka, and to examine whether the severity of alcohol-related problems (ARPs) in men and presence of alcohol-related domestic violence are associated with MDD among these women.

**Method:** In a cross-sectional study, ARPs among men were assessed using Alcohol Use Disorders Identification Test (AUDIT) questionnaire filled in by men, and domestic violence and husbands' drinking pattern data obtained from the women. MDD among the women was ascertained using the Structured Clinical Interview for DSM–IV Disorders for major depression. Using logistic regression we examined whether age, past history of depression, different indices of ARPs and domestic violence were associated with current MDD among the women.

**Results:** Point prevalence of MDD in the sample was 33.3% (95% CI: 25.93, 40.73%). Once adjusted for other factors, morning drinking of the spouse (odds ratio = 4.11, 95% CI: 1.25, 13.47; *P* = 0.019) and increasing age (odds ratio = 1.05, 95% CI: 1.01, 1.09; *P* = 0.003) significantly increased the odds of MDD. Being subjected to domestic violence/arguments also had a trend to be associated with MDD among women, but was not significant (odds ratio = 2.29, 95% CI: 0.95, 5.48; *P* = 0.062).

**Conclusion:** The prevalence of MDD among spouses of men who use alcohol is markedly higher than that has been observed among Sri Lankan women in previous studies. The prevalence of MDD in women seems to increase when their husbands are morning drinkers, and with increasing age.

## INTRODUCTION

### Alcohol-related problems in Sri Lanka

According to the ICD-10 Classification of Diseases, alcohol-related problems (ARPs) include harmful use and alcohol dependence syndrome. The AUDIT (Alcohol Use Disorders Interview) has also promoted the wide use of the category ‘hazardous use', including in Sri Lanka where ARPs are a common health issue (De Silva *et al.*, 2008)*.* An early epidemiological survey reported the prevalence of ‘alcoholism’ among Sri Lankan men over 25 years of age to be 2.9% (Samarasinghe *et al.*, 1987). However, this may have been an underestimate due to the tendency of these patients to under-report the extent and problems associated with their drinking especially in the presence of their family members as was the case in the above study. Buddhist religion—in which alcohol consumption is considered immoral—also contributes to guilt and under-reporting of alcohol use. It is also possible that the prevalence of ARPs has increased along with increased use of alcohol associated with change in cultural values over the last few decades (Samarasinghe, 2006). A more recent study conducted in Sri Lanka shows the prevalence of alcohol consumption (at least one drink during the preceding month) to be 32.9% in an urban sample and 20.8% in a rural sample (De Silva *et al.*, 2009). In this study 5.16% of the urban sample and 1.46% of the rural sample reported that they consumed alcohol daily. According to the 2006 Global Status report on alcohol, 2.4% of the whole adult population and 4.9% of the males in Sri Lanka were ‘heavy episodic drinkers’. Ninety-four per cent of alcohol is consumed in the form of spirits (WHO, 2006).

About 10–20% of people who drink alcohol excessively develop cirrhosis, and there are correlations in a population between rates of liver cirrhosis and mean alcohol consumption. Therefore deaths from cirrhosis can be considered as a reliable indicator of problem drinking in a country (Gelder *et al.*, 2005). Census and Statistics department in Sri Lanka, reported that in 2005, cirrhosis mortality rate to be 33.4 per 100,000 males (Department of Census and Statistics, 2010)—one of the highest in the world—which compares with 14.1 in the UK and 28.1 in France (Leon and McCambridge, 2006).

### Effects of alcohol-related problems on family wellbeing

ARPs could affect the wellbeing of one's spouse and the family. According to Halford *et al.* (1999), women in that situation may face domestic violence and threats of violence, emotional and sexual abuse, lack of confiding relationships, social isolation due to humiliation, financial hardships, chronic psychiatric or physical problems of the drinking husband directly related to alcohol abuse or secondary to alcohol abuse (e.g. depression), and marital disharmony which in turn could lead to perpetual psychological distress, and a risk of developing depression which in unhappily married women is ∼25 times than that in happily married women (Weissman, 1987). A prolonged increase in marital arguments is the most frequent life-event reported as preceding the onset of depression in married women (Halford *et al.*, 1999). According to a recent study conducted in India 43% of spouses of men with ARPs had major depressive disorder (MDD) and the depression had significant correlation with the severity of the ARPs measured by alcohol dependence data and an inventory of consequences (Kishor *et al.*, 2013).

Campbell (2002) identifies depression and post-traumatic stress disorder as the major psychiatric morbidities in the victimized women, the risk of depression being even higher than among the victims of childhood sexual abuse (Campbell, 2002). Similarly, Medina-Mora reports a prevalence of depression of 8% among wives of alcoholic partners, which further increased to 25% when the women were victims of domestic violence (Medina-Mora, 2001).

Despite the likely high prevalence of ARPs among men in Sri Lanka, there is no research to date on MDD among their spouses. Our aim was to estimate the prevalence of MDD among spouses of men who use alcohol in rural Sri Lanka and to examine the association of the severity of alcohol-related problems (ARPs) in men and presence of alcohol-related domestic violence with MDD among these women.

## METHODOLOGY

### Study design and setting

The present study was conducted in conjunction with an interventional study that was started in 2008 by the Department of Psychiatry, Faculty of Medicine, University of Peradeniya (Siriwardhana *et al.*, 2013). We selected two villages (Handabowa and Handapaldeniya) separated by a distance of 15 km and of comparable size, population and socio-economic backgrounds. These villages were in separate local government divisions of the Central Province and were selected because information gathered showed that these two villages had a higher number of illicit distilleries and higher consumption of alcohol than other villages in the divisions. Approval for the study was granted by the Ethics Committee of the Faculty of Medicine, University of Peradeniya, Sri Lanka. Written consent was taken from all women before the clinical interviews. The women who were detected to have MDD were directed to the nearby hospital at their own discretion.

### Participant recruitment and clinical assessment

The villages had an estimated population of around 1000 and 246 identified adult men. Baseline data on alcohol use and ARPs were collected from men using the Sinhala translation of the Alcohol Use Disorders Identification Test (AUDIT) (De Silva *et al.*, 2008) as a part of a wider study (Siriwardhana *et al.*, 2013).

Of the 246 men to whom AUDIT was administered, 44 answered that they had never consumed alcohol, while 202 (82%) used alcohol to different extents. Of those who used alcohol, AUDIT scores estimated the level of drinking as low risk (0–7) in 37 (15% of the total sample) men, hazardous (8–15) in 73 (30%), harmful (16–19) in 53 (21%) and dependent (20 or above) in 39 (16%). Further details of the drinking patterns of this sample have been reported earlier (Siriwardhana *et al.*, 2013). For the present study, we considered those who had AUDIT scores of 8 or more as having possible ARPs whereas those who had AUDIT scores <8 were not to have possible ARPs. The above stratification has been shown to have good sensitivity and specificity (Conigrave and Hall, 1995; Babor *et al.*, 2001). For data analysis, we also defined ‘probable dependence’ in two methods (as recommended by Babor *et al.*, 2001): first, those with AUDIT score of 20 or more, and second, a score of 4 or more on questions 4, 5 and 6 (i.e. dependence domain) of the questionnaire.

Of those 202 men who used alcohol, 168 were married to or cohabiting with women. For the present study we visited each of these 168 households to recruit the eligible women. The interviewer of female participants was a female psychiatrist (first author). (Males in the AUDIT study had been interviewed by a trained male postgraduate student—third author). Up to three visits were made to each household to contact the prospective participants while they were home and to interview them in the absence of their spouses to minimize the biases of responses due to any actual or perceived threats of violence or intimidation. Five women were excluded either because the women consumed alcohol themselves, had psychotic illnesses or had recent bereavement. Out of the 163 remaining potential subjects, 156 women could be contacted and recruited for the study after three home-visits (response rate = 95.7%). Seven women in the recruitment list could not be contacted. The interviews were conducted by the first author (DA) a psychiatrist, in Sinhala the first language of the participants. The data were collected from January to April 2009.

In addition to the AUDIT scores derived from interviewing husbands, we directly questioned the women whether their husbands are aggressive/argumentative or violent towards them, whether they consume alcohol in the morning (‘eye opener’). We considered these latter two indicators as two important and objective measures that can be obtained from an observer other than the alcohol consumer himself, which suggested domestic violence and alcohol dependence respectively.

The outcome measure of interest was MDD among women as assessed by Structured Clinical Interview for DSM IV Disorders (SCID, Biometrics Research Department, Columbia University, NY, USA). The SCID-IV criteria check for nine symptoms (viz. depressed mood, markedly diminished interest or pleasure, weight loss/gain or reduced/increased appetite, insomnia/hypersomnia, psychomotor agitation/psychomotor retardation, fatigue/loss of energy, feelings of worthlessness/excessive or inappropriate guilt, diminished ability to think/concentrate or indecisiveness and thoughts of death, suicidal ideation, attempt or plan). According to SCID-IV criteria each symptom is counted if it persisted at least over a 2-week period within last 4 weeks preceding the assessment (First *et al.*, 2002). A given participant was considered having an episode of MDD, if she reported experiencing at least five of the nine symptoms, of which one should be either depressed mood or markedly diminished interest or pleasure, during the same 2-week period. Once SCID was completed, a clinical interview was conducted to ascertain whether they had a history of depression in the past, looking for the presence of persisting symptoms for at least for 2 weeks.

### Data analysis

A series of simple logistic regression analyses were initially performed to examine whether different independent variables are significant predictors of current MDD among the women. These variables included: (a) demographic factors viz. age, village and monthly family income category, (b) different measures of ARPs and/or dependence in men viz. raw total AUDIT score, presence of hazardous or harmful use or dependence (based on, i.e. total AUDIT score ≥8 vs. <8) and presence of probable dependence according to two methods recommended by Babor *et al.* (2001) (i.e. AUDIT total score ≥20, and a score of more than 0 on dependence domain questions 4, 5 and 6) and (c) factors reported by women: past history of depression, and presence of morning drinking and domestic violence of their spouses. The factors that had a significant association with MDD in the simple regression analyses were entered in to a multiple logistic regression model. As recommended by Hosmer and Lemeshow, a less conservative level of significance (*P* < 0.25) was set in choosing factors to be entered into the multiple regression model (Hosmer and Lemeshow, 2000). All other statistical comparisons were interpreted at a cut-off *P*-value of 0.05. Data were analysed using the statistical software STATA Version 11.1.

## RESULTS

We recruited 71 women (age range: 19–74 years, mean: 37.7, SD: 11.6) from Handabowa, and 85 women (age range: 22–73 years, mean: 39.7, SD: 12.1) from Handapaldeniya. The average age was similar between the two groups of women (*P* = 0.292). Of the total number of 156 women only 4 were regular employees (one each: school teacher, clerk, worker in a retail shop, tailor). Of the rest, 25 reported that they sometimes work as casual labourers, but not on a regular basis. The remaining 127 (81.4%) were unemployed. The sample characteristics are summarized in Table 1.

**Table 1.** Sample characteristics

	Handabowa (*n* = 71)	Handapaldeniya (*n* = 85)	Significance (*P*)
Mean age (SD)	39.4 (11.9)	37.8 (11.7)	0.292^a^
Monthly family income category (% of total no.): Sri Lankan Rupees			0.012^b^
0–4999	6.1	1.8	
5000–14,999	38.8	69.1	
15,000–24,999	51.0	29.1	
≥25,000	4.1	0.0	
Past history of depression (%)	11 (15.5)	14 (16.5)	0.426^b^
Depression diagnosed according to SCID criteria (%)	26 (36.6)	26 (30.6)	0.868^b^

^a^Student's *t*-test.

^b^Chi-square test.

The point prevalence of MDD in the study sample was 33.33% (95% CI: 25.93–40.73%). There was no significant difference in prevalence of MDD between the two villages in the study sample (odds ratio = 1.31, 95% CI: 0.67–2.56, *P* = 0.868).

Eleven women in Handabowa (15.5%) and 14 women in Handapaldeniya (16.5%) reported a past history of depression. Unexpectedly, past history of depression, as reported by the subjects, did not emerge as a significant predictor of current diagnosis of MDD (OR: 1.15, 95% CI: 0.47–2.82, *P* = 0.758).

Of the factors entered into simple regression models (viz. age, village, monthly income category, past history of depression, different measures of ARPs or dependence in men, and morning drinking and domestic violence as reported by women), increasing age (unadjusted OR = 1.06, 95% CI: 1.03, 1.09, *P* = 0.002), morning drinking (unadjusted OR = 5.83, 95% CI: 1.92, 17.69, *P* = 0.002) and domestic violence (unadjusted OR = 3.05, 95% CI: 1.38, 6.75, *P* = 0.006) turned out to be significant predictors of the present episode of depression, and thus were entered into multiple regression model (Table 2) [‘Probable dependence’ (as indexed by an AUDIT score of 20 or more) did not have a significant association with MDD among women (OR = 2.22, 95% CI: 0.68–7.28, *P* = 0.185), but passed the cut of significant limit (i.e. <0.25) set for candidate variable suitable to enter to multiple logistic regression modelling. ‘Probable dependence’ was strongly correlated with morning drinking as reported by the women (OR = 12.34, 95% CI: 2.99–50.96, *P* = 0.001). and thus we did not enter both dependence and morning drinking into the same model, but ran two MLR models: first with age, domestic violence and dependence as predictor variables and the second with age, domestic violence and morning drinking (reported by women) as predictor variables. The model with ‘probable dependence’ was not significant (*P* = 0.056) and was weaker (pseudo *R*^2^ = 0.0668), while the second (with age, domestic violence and morning drinking) was significant. Therefore we report the second model in detail in the manuscript.]. There was a significant correlation between men's morning drinking as reported by the men (for AUDIT question number 6) and by the women (chi squared: 10.975, *P* = 0.027).

**Table 2.** Factors associated with current episode of depression in univariate and multiple logistic regression analyses

Predictor variable	Univariate analysis		Multiple logistic regression	
	Unadjusted OR (95% CI)	*P*-value	Adjusted OR (95% CI)	*P*-value
Village	1.31 (0.67, 2.56)	0.427	–	–
ARPs (AUDIT score ≥8)	0.84 (0.33, 2.15)	0.715	–	–
Depression in the past	1.15 (0.47, 2.82)	0.758	–	–
Age (per 1 year increase)	1.06 (1.03, 1.09)	0.0002	1.05 (1.02, 1.09)	0.003
Domestic violence	3.06 (1.38, 6.75)	0.006	2.29 (0.96, 5.48)	0.06
Morning drinking in spouse	5.83 (1.92, 17.69)	0.002	4.12 (1.26, 13.47)	0.019

ARPs of the husband as indexed by an AUDIT score of 8 or more (unadjusted OR = 0.84, 95% CI: 0.33, 2.15, *P* = 0.715), or the past history of depression reported by women (unadjusted OR = 1.15, 95% CI: 0.47, 2.82, *P* = 0.758) were not significant predictors of current MDD. However, there was an intercorrelation between presence of ARPs and domestic violence. Men with ARPs were 2.7 times more likely to get into arguments or commit domestic violence (odds ratio = 2.72, 95% CI: 1.01, 7.36, *P* = 0.048).

The final multiple regression model containing age, morning drinking and domestic violence explained 15% of the variation in occurrence or non-occurrence of MDD (pseudo *R*^2^ = 14.87, *P* < 0.0001). Once adjusted for the other significant predictors in the multiple regression model, each 1-year increase of age increased the odds of MDD by 5% (adjusted OR = 1.05, 95% CI: 1.02–1.09, *P* = 0.001), and those whose husbands were morning drinkers were 4.1 times (adjusted OR: 4.12, 95% CI: 1.26, 13.47, *P* = 0.019) more prone to MDD. Being subjected to domestic violence/arguments also tended to be associated with MDD among women, but was not statistically significant once adjusted for other factors (odds ratio = 2.29, 95% CI: 0.95, 5.48; *P* = 0.062). When we examined the subgroup of women whose husbands had ARPs (i.e. AUDIT score of 8 or more), prevalence of MDD was 40.5% when they were subjected to domestic violence while the prevalence was 18.5% when they were not subjected to domestic violence.

## DISCUSSION

The association between MDD in women and the alcohol use among their spouses has to be interpreted in the context of our study population, which is a rural community, in which a large majority of men consumed alcohol and had ARPs to different extents, and a large majority (81%) of the women were not employed and thus financially dependent. The community we studied is not representative of the general population in Sri Lanka in terms of alcohol consumption and associated problems and thus has limitations in generalizability. Eighty-two per cent of the men in the study villages consumed alcohol while 16% were possibly dependent. These figures are much higher than that observed in other local studies. In comparison, a recent study from a different rural population reports the prevalence of alcohol consumption (at least one drink in the previous month) to be 20.8%, and only 1.46% consumed alcohol daily (De Silva *et al.*, 2009). An early survey reported the prevalence of ‘alcoholism’ to be 2.9% in a semi-urban community (Samarasinghe *et al.*, 1987).

Only 2.6% of the women in our sample had an employment with a regular income. While unemployment itself is a risk factor for depression, it is possible that the fact these women have to financially dependent on their husbands can compound the helplessness and psychological stress associated with their husbands' drinking behaviour thus contributing eventually to depression. Thus, one methodological limitation of our study is that we could not extend our interview to wider socio-demographic domains which meant we could not examine the influence of some additional socio-demographic factors that could contribute to depression in those women (e.g. level of education, the degree of support from the extended family, the number of children on the family, etc.).

In spite of these limitations, the results show patterns not documented hitherto in Sri Lanka. In this rural population, one in three women whose husbands used alcohol had MDD. These figures are markedly higher than that has been reported previously among Sri Lankan women in the general population—for example, a community-based survey conducted in Colombo—the capital of Sri Lanka—estimated a lifetime prevalence of depression in women to be 8.1% and point prevalence of 1.78% (Ball *et al.*, 2010). The urban-rural distinction and associated socio-demographic factors may have contributed partially to this discrepancy, but the difference between the two studies is too large to be explained by the demographic factors alone. The prevalence of MDD observed in our study is even higher than that reported among women from other high-risk populations in Sri Lanka viz. tsunami survivors (19.1%) (Hollifield *et al.*, 2008), civilians affected by war (27%) (Somasundaram and Sivayokan, 1994). The prevalence that we observed was also markedly higher than the estimates reported in a previous Mexican study where women living with men having ARPs had a prevalence of depression of 8% (Medina-Mora, 2001).

In the regression analysis conducted based on a number of possible risk factors, the habit of morning drinking (i.e. need for an ‘eye opener’) among their spouses, and increasing age emerged as significant risk factors for MDD among women. Wives of morning drinkers were four times more prone to MDD. The severity of ARPs of men (as indexed by the AUDIT score of 8 or more) had no significant association with MDD among women, while there was a statistical trend for the presence of arguments/violence to have an association with MDD.

In the subgroup of women whose husbands had ARPs (i.e. AUDIT score of 8 or more), prevalence of depression among the women who were not subjected to domestic violence was 18.5%, but was 40.5% among those who were subjected to domestic violence. The findings of previous studies seem to be consistent with these results (Medina-Mora, 2001; Kahler *et al.*, 2003; Homish *et al.*, 2006). Medina-Mora *et al.* (2001) reported a Mexican household prevalence of MDD among spouses of men with ARPs as 8%; this increased to 25% among those subjected to domestic violence by their husbands, and the risk of MDD increased further if the abused spouse was pregnant. Similarly, Kahler *et al.* (2003) observed psychological distress among women to be significantly associated with the presence of physical violence, but not with the severity of alcohol problems in their partners (Kahler *et al.*, 2003). Homish *et al.* (2006) also report that the frequency/amount of drinking by the male partner *per se* had no bearing on ‘depressive symptomatology’ of their women (Homish *et al.*, 2006). Although we did not find ARPs of the men to be an independent a predictor of depression among the women, as has been reported in the above studies, Dawson *et al.* (2007) observed alcohol use disorders (classified according to DSM IV criteria) among men to be an independent risk factor for depression in partners even after adjusting for the presence of domestic violence (Dawson *et al.*, 2007).

Increased point prevalence of MDD with advancing age is noteworthy. In our sample, the odds of MDD increased by 5% with each increasing year of age. There is evidence that the association between marital distress and major depression increases in magnitude with increasing age (Whisman, 2007). Therefore, rather than the effects of advancing age *per se*, increasing prevalence of MDD in women may be an adverse effect of long term cohabitation with an alcohol dependent spouse and who tend to get violent towards the woman.

In conclusion, our study shows that in rural communities of Sri Lanka with high prevalence of alcohol consumption, on average one in three women whose husbands use alcohol has MDD. The risk of MDD increases with increasing age and when their husbands are morning drinkers. One interpretation of these results is that the women who are married to men who are dependent on alcohol and resort to domestic violence progress into depression, and the risk increases as the couple stay together for a longer period of time. However, the cross-sectional design of this study precludes establishing such direct cause and effect relationship, and the interrelationship between those factors is very likely to be more complex and perpetuating. Particularly in this type of rural, economically disadvantaged communities, social and economical stressors may lead to concurrent increase in alcohol consumption among men and depression among women. Furthermore, it is also possible that depression of women contribute to increased alcohol consumption among men setting up a vicious cycle. Despite the limited insights into the mechanisms, our findings raise important clinical and public health implications. Given the high prevalence of depression among spouses of men who use alcohol, health professionals who work with the men who use alcohol in these communities should be aware of the possibility of depression in the spouse and try to manage the family unit as a whole. Obtaining a concurrent history from the spouses of those men would help to understand the perception of those women about the alcohol consumption of their husbands, determine the impact of alcohol use on the family, and elucidate the factors such as dependence, increasing age, domestic violence that possibly mediate depression among the women. Public health interventions aiming to cut down alcohol use in these communities should not only address the alcohol habits of men, but also depression among their spouses.

## FUNDING

This study was partly supported by the South Asian Clinical Toxicology Research Collaboration, which is funded by the Wellcome Trust/National Health and Medical Research Council International Collaborative Research Grant GR071669MA. The study sponsor had no role in study design, data collection, data analysis, data interpretation or writing of the report. Funding to pay the Open Access publication charges for this article was provided by the Wellcome Trust.

## CONFLICT OF INTEREST STATEMENT

None declared.
